# Harm reduction in severe and long-standing Anorexia Nervosa: part of the journey but not the destination—a narrative review with lived experience

**DOI:** 10.1186/s40337-024-01063-3

**Published:** 2024-09-12

**Authors:** Edwin Birch, James Downs, Agnes Ayton

**Affiliations:** 1 Oxford Health NHS Foundation Trust, Oxford, OX4 4XN UK; 2 Expert by Experience and Independent Researcher, Oxford, UK

**Keywords:** Anorexia Nervosa, Severe and enduring Anorexia Nervosa, Eating disorders, Harm reduction, Treatment

## Abstract

Questions remain about the best approaches to treatment for the subset of patients with severe and long-standing Anorexia Nervosa, commonly described in the literature as “Severe and Enduring Anorexia Nervosa.” When discussing the optimal strategies and goals for treating this group, there is uncertainty over whether to focus on refining current treatment methods or exploring alternative approaches. One such alternative is “harm reduction,” which has generated a wave of positive interest from patients and clinicians alike because of its emphasis on individual autonomy, personal goals and quality of life. While harm reduction can provide an attractive alternative to seemingly endless cycles of ineffective treatment, this narrative review builds on previous work to highlight the inadequate terminology and possible dangers of considering harm reduction as the endpoint of treatment. In conjunction with perspectives from a lived experience author, we consider wider contextual and ethical issues in the field of eating disorders, which should inform the role of harm-reduction approaches in this patient group.

## Abstract (plain language summary)

One model of treatment for patients with severe and long-standing Anorexia Nervosa is termed “harm reduction”, which moves away from traditional treatment aimed at full recovery and weight gain. This approach instead prioritises quality of life, giving patients greater control over their care. Harm reduction remains ethically controversial due to concerns about unaddressed malnutrition and issues of consent for this subset of patients. This review examines the inadequacies in how severe and long-standing Anorexia is defined, alongside exploring the ethical concerns of harm reduction with lived experience from one author.

## Introduction

There is a need for alternative treatment modalities to alleviate the suffering from repeated cycles of failed conventional treatment in severe and long-standing Anorexia Nervosa, with “Harm reduction” being one such approach which has gained considerable attention in recent research [1, 2].

At its core, it has been well summarised by Tumba et al. as a strategy that: “*should help the patient maintain a weight that balances quality of life but is below ideal weight range and still associated with medical risks*” [3] p 17.

They, among others, posit that harm reduction is a more ethical pathway which avoids progression to involuntary treatment methods, which may be necessary for the non-consenting patient who is severely ill [4]. By embracing a shared personal treatment goal that sits outside of conventional recovery (i.e. abandoning full weight restoration and medical stability), clinicians may promote a greater degree of patient autonomy and focus on quality of life. Furthermore, even though a personalised treatment goal can be at odds with full recovery, a recent commentary from Bianchi et al. did not find ethical concerns a barrier to the use of harm reduction in eating disorders (ED) as a whole [5].

In addition to pursuing acceptable therapeutic objectives for patients, it is essential to examine the concept of harm reduction, as data suggest that this severe and long-standing condition may impact approximately 20–30% of those diagnosed with anorexia nervosa (AN) [2, 6].

## Aims

This narrative review examines the current literature on harm reduction as a treatment strategy and, with reference to three core ethical concerns, benefits and harms.

## Methods

A literature search was conducted to identify relevant papers for the present review. PubMed, Google Scholar, and Ovid MEDLINE databases were all searched between 1995 and 2024 with additional references drawn from relevant articles. Search criteria for PubMed and Ovid: (Anorexia OR Eating disorders OR Severe and enduring) AND Harm Reduction. Search criteria for Google Scholar: Anorexia AND “Harm reduction” NOT addiction NOT opioid NOT opiate NOT alcohol NOT bulimia. A total of 341 abstracts were initially retrieved and reviewed, of which 23 were identified as relevant to the subject material. We also include lived experience perspectives on this controversial issue.

### What is “severe and enduring” Anorexia?

The complexity of harm reduction interventions is exacerbated by a lack of agreement regarding the patient population for which it is deemed appropriate. Nevertheless, many NHS services have developed local severe and enduring eating disorder (SEED) pathways which refer to harm reduction as a guiding principle of care, even though this category of patients is not defined by diagnostic systems. The latest editions of the ICD 11 and DSM-5 do not include diagnostic categories based on the length of illness. Both define the severity of Anorexia based on body mass index (BMI) as an indicator of the risks associated with malnutrition [7, 8].

For chronic presentations, a diverse array of labels is in use, the most common being "*Severe and enduring Anorexia Nervosa*” (SE-AN), with other entities such as “*chronic-intractable*”, “*enduring and serious*”, and “*end-stage*” appearing less frequently [9, 10].

The SE-AN construct remains the most widespread to date, however patients prefer the term “severe and long-standing” in its place [11] as at the time of writing there remains no accepted definition for the term among research groups [12, 13]. Hay et al. proposed a set of clinical criteria for defining SE-AN in 2018, including a triad of low body mass index (BMI) (with cardinal AN psychopathology), an illness duration of at least 3 years, and a failure of 2 evidence-based treatments [14]. However, on closer examination, there still exists a wide variation about the duration of illness required to warrant a”severe and enduring” classification, alongside variable definitions of “failed cycles of treatment”- both of which are the most common features assumed to delineate this subgroup [10].

The heterogeneity in definitions of the “enduringness” and “severity” components of SE-AN speaks to the rather arbitrary adoption of these criteria between research groups (Table 1).

**Table 1:** Examples of research papers on Severe and Enduring Anorexia in the literature

Authors	Year	Article Type	No. of participants	Duration of illness (years)	Definition of severity	Previous evidence-based treatment
Arkell and Robinson	[47]	Descriptive study	11	> 10	NS	NS
Daansen and Haffmans	[48]	Observational study	5	≥ 5	NS	Yes
Touyz et al.	[20]	Randomised Controlled Trial (RCT)	63	≥ 7	NS	Yes
Robinson et al.	[46]	Qualitative study	8	20–40	NS	NS
Calugi et al.	[40]	Longitudinal outcome study-inpatient	31	> 7	NS	Yes
Raykos et al	[39]	Outpatient treatment	134	≥ 7	NS	NS
Hay and Touyz	[14]	Editorial	n/a	> 3	NS	Exposure to 2 evidence-based treatments
Bianchi et al.	[5]	Commentary	n/a	≥ 6	NS	NS
Zhu et al	[23]	Cochrane review of RCTs	242	≥ 3	NS	Yes
Tumba et al	[3]	Commentary	n/a	> 10	NS	Yes
Robison et al.	[45]	Retrospective cohort	782	> 30 years of age	NS	Yes

*NS* not specified: The study did not define the term or provide specific data for the category

Yes: previous evidence-based treatments were referenced in the study

Duration of illness: years

Furthermore, although studies have identified neurochemical changes in the brain during severe disease [15], biological markers for objectively identifying SE-AN are lacking [16].

Table 1 summarises the definitions of SE-AN in the literature between 2008 and 2024. These studies used a variety of methodologies, including observational, longitudinal, qualitative, and randomised controlled trials (RCTs), with participant numbers ranging from 5 to 782 and illness durations ranging from 3 to > 40 years. Furthermore, there are inconsistencies in the use and reporting of evidence-based treatments. Whilst studies by Daansen and Haffmans [48], Touyz et al. [20], and Zhu et al. [23] explicitly mention the use of evidence-based treatments, others, such as Arkell and Robinson [47] and Robinson et al. [46], do not. Moreover, the severity of EDs is largely undefined across studies, which further complicates the interpretation of treatment outcomes. These disparities highlight the need for more standardised reporting and methodological rigour in ED research to better assess the efficacy of treatments and interventions [1].

### The case for harm reduction

Hay and Touyz conducted the first systematic review of treatments specific to SE-AN in 2012 and identified only 12 studies between 1997 and 2011, with non-specific overall findings. These indicated that specialist psychotherapy modalities such as Cognitive Behavioural Therapy for Anorexia Nervosa (CBT-AN) and Enhanced Cognitive Behavioural Therapy (CBT-E) may be preferable to “treatment as usual”, which comprised of a range of modalities. The comparisons made were hampered by high study heterogeneity, but it is noteworthy that even at that time, the authors called for a move towards examining efficacy of harm minimisation beyond weight restoration as the primary end goal [17].

At the time of writing, no RCTs have examined harm reduction treatments for severe and long-standing AN, highlighting the ongoing need for explicit research in this area. Apart from the challenges of scant data, we anticipate difficulties unique to measuring the “success” of harm reduction, given discrepancy in individual patients’ own agreed treatment plan. Measures of success in this area could be further limited by disagreement on the best parameters to target; conflicts may arise over emphasis on patient led reductions in psychopathology vs. service led outcomes, such as mortality and inpatient admissions. The analysis on whether such effects are mutually exclusive remains outstanding.

Despite the lack of RCTs, we do identify research exploring interventions with a harm reduction approach, though they still have limitations and often use anecdotal data in the form of individual case series.

One prominent example is the Community Outreach Partnership Program (COPP), spearheaded by Williams et al. who found that utilising a harm reduction approach resulted in a significant decrease in ED symptoms and a modest increase in BMI (1.24 point increase across all patients included in the study). Interestingly, the primary outcome of self-reported “quality of life” showed no statistically significant difference, although only 15 patients with AN were included [18]. Additionally, Yager et al. have been vocal about the positive experiences of harm reduction, where they coin the phrase “compassionate witnessing” as a beneficial therapeutic stance to engage with patients with severe and long-standing AN [19].

We also consider the literature related to treatments which share the goals of a harm reduction approach, either as implicit or explicit component. For example, given that harm reduction prioritises an increase in quality of life and reduction in invasive interventions, it is noteworthy that an RCT of 63 patients using Specialist Supportive Clinical Management (SSCM) and CBT-AN showed significant improvements in eating disorder symptomatology and health-related quality of life, which persisted during a 1 year follow up period with a 76% completion rate [20].

Cognitive remediation therapy (CRT) was also explored by Dingemans et al. showing an improvement in eating disorder related quality of life in a mixture of chronic and acute patients [21]. 

Although there has been a growing demand from both clinicians and patients for the integration of harm reduction approaches in the treatment of severe and chronic AN [16, 22], there is currently a scarcity of concrete evidence regarding their efficacy and there is no clear consensus on what constitutes effective implementation and positive outcomes in this context. It is telling that the recent 2023 Cochrane review into psychological therapies for SE-AN, full weight restoration is still maintained as the primary outcome, with features of quality of life and eating disorder symptomatology remaining secondary [23].

### Is harm reduction ethical?

We highlight three core concerns in the scenario where harm reduction is considered the focus of treatment for severe and long-standing AN. We have incorporated narrative reflections from lived experience of this condition to centralise the ways in which these theoretical understandings may play out in clinical settings and individual lives. This, integrated with an overview of the evidence, will inform our conclusions regarding the use of harm reduction in modern practice.

## Are we truly reducing harm? Dangers inherent in ignoring the physiological consequences of malnutrition

Harm reduction arose from addiction treatment in the 1980s, when it was recognised that for many patients, the destructive trail of drug-seeking behaviour, especially for opioids, could be offset to a degree by medically supervised provision of drugs [24]. A similar parallel has been drawn with severe and long-standing AN, whereby some of the social cost of repeated inpatient admissions and medical complications may be mitigated by accepting, in conversation with the patient, a lower BMI target. However, recent work has shown that the model derived from addiction may not be readily comparable, most pertinently because there is a misplaced assumption (or perhaps overlooked reality) that a low BMI target will offset the harms of malnutrition [9, 16]. Furthermore, harm reduction strategies are supported by evidence of improved outcomes in the addiction field, whilst similar studies have not been conducted in the eating disorder field [25].

Malnutrition, regardless of its extent, has deleterious effects and can cause organ damage both in the short and long term. While most of the consequences are reversible with appropriate weight gain, it is important to recognise the potential for long-term damage [26, 27]. All organ systems are affected, most notably the impact on the cardiovascular system can be life threatening [28]. The consequences of poor nutrition on bone density may worsen over time, with the potential to remain unaddressed (or unchecked) as part of a harm reduction strategy [29].

Structural changes in the brain due to malnutrition are well-documented and can be largely reversed by weight restoration [30]. A recent prospective analysis of 1648 patients by the ENIGMA group, including healthy controls, acutely underweight, and partially weight restored AN patients, found widespread reduction in cortical thickness, subcortical volumes and cortical surface area which was closely related to BMI [31]. Whilst there is significant improvement in all three parameters in the partially weight restored group (n = 251), they are still not at the level of healthy controls, highlighting the need for full weight restoration to restore pre-morbid neural architecture.

Whilst the relationship between structural brain differences and psychopathology remains uncertain, Walton et al.’s suggestion of a possible link between effects in the superior and inferior parietal gyrus to alterations in attention and “body-environment integration” is intriguing and complements a growing body of work on cognitive deficits in individuals with extremely low BMI [15].

When considering dangers of malnutrition states, we must also be cognizant of the rapidity of decline in certain cases, even amongst patients who have thus far managed to sustain severe and long-standing illness. Some patients can exhibit remarkable resilience in the face of severe malnutrition, leading clinicians to erroneously assume their medical stability [16]. Arrythmias and severe hypoglycaemia can send superficially “medically stable” patients into sudden and sometimes fatal decline; indeed, this risk of rapid decline has been noted in qualitative accounts of patients experiencing living with an extremely low BMI [32].

Considering the significant impacts and risks of persisting illness, harm reduction in these patients may therefore be a misnomer. Whilst involuntary admissions may be avoided in the short term by agreeing treatment goals that deprioritise weight status, the long-term physical and mental health consequences, even at marginally lower baseline BMI targets, must lead us to question whether harm is really being reduced *enough*.

In author JD’s lived experience of severe and long-standing AN, considerable physiological and psychosocial harm was incurred by the maintenance of a very low BMI for approaching a decade (Box 1).

**Box 1:** Author JD reflects on his experiences of malnutrition

“Whilst clinicians were not so concerned when I was stabilised from recurrent medical crises, staying a very low BMI limited my ability to participate in life and continued to erode my physical health over time, with serious lasting consequences. I feel that, throughout a lot of my treatment, I was never given the chance to experience what it was like to be in an adequately nourished body, and to realise that I did not have to live with enduring suffering to the degree that I did when very underweight. Many of the harmful symptoms I experienced resolved with nutrition, yet I was told that this was just the way I was and that I would likely live with anorexia in some form forever. It wasn’t ever going to be a nice or easy experience to recover my health, but I would rather have been able to do it sooner, with fewer consequences that I still live with today”	

## To consent or not to consent? An over-reliance on presumed patient capacity

The issue of capacity in severe and long-standing AN illness (see [33]) is a major ethical barrier to harm reduction. In the research for this review, we found every proponent of harm reduction to highlight the importance of informed consent before pursuing this approach, with the default assumption that patients are able to retain capacity for such decisions [5, 18, 19].

However, as eloquently summarised by Geppert amongst others, when dealing with severe and long-standing AN, we must call into question the validity of this consent regarding treatment decisions, particularly the ability to weigh up information [9, 34].

We should also be cautious of the capacity of patients consenting to a treatment plan suggested by a clinician (i.e. offered as “medical advice”). Individuals with lived and living experience of severe and long-standing AN have raised concerns that harm reduction approaches may be seen by patients as a way to engage in a form of treatment without the expectation of substantial behaviour change, thus “allowing” the perpetuation of illness [32, 35]. There may also be a possible role for unconscious motivations in incentivising a less resource-intensive option for specialist services that are so under-resourced as to only be able to offer their patients a form of “managed decline”, rather than evidence-based and recovery-focused treatment [36].

Author JD (Box 2) reflects on some of the complexities of making decisions regarding treatment.

**Box 2:** Author JD reflects on his experience with capacitous decision making

It might be easy to say this with relative hindsight, but I do not believe that I had the capacity to make decisions about my care when I was seriously malnourished. One example is how I have had profound, refractory hypokalaemia (low potassium). This most recently happened over many months during a period of treatment which was not focussed on eating disorder symptoms, but on my quality of life and relationships Low potassium levels have in themselves made me feel suicidal, dysregulated, and unable to think properly—all of which have resolved rapidly with the replenishment of this vital nutrient. I have really struggled with medical admissions for this, which I have found particularly distressing as I've had past experiences where I have had my care mismanaged, not had my co-occurring conditions and neurodivergence considered, and experienced the stigma that so many patients with eating disorders are persistently met with Even when I have told staff straight away that I will want to discharge myself, will need support to stay, and do not want to be allowed to discharge myself, when the time comes that I feel too distressed to stay in the hospital for treatment, I have always been told that I have capacity to make that decision. I may be able to eloquently explain my cardiovascular risk even when I have life-threateningly low potassium. I may, in part, want to refuse treatment. But this is not because it is what I really want. I want staff to support me in making it bearable, to reduce the harmfulness of the experience, but only so that I can get the treatment Looking back, I know that I have been allowed to make decisions based on what are actually features of my illness, rather than by engaging the part of me—often obscured—that has wanted to be well. Sometimes, I resent this. I am grateful for the times when I have been met with a firmer approach from staff, who have not deferred to my ‘rights’ and my ‘patient choice’ when I have been cognitively impaired by my physiology. Just because I have rights does not mean that I always have been right. Just because staff have been forceful with me at times doesn’t mean that they’ve done so without also showing me that they are ultimately on my side. Ultimately, I am grateful for having my life saved when, in those moments, I did not want it to be—even if it was an incredibly difficult experience at the time.”	

## Is harm reduction an admission of futility?

Harm-reduction approaches are, by definition, closely aligned with the concept of futility in psychiatry, which remains a highly controversial area [37]. In one sense, by abandoning the traditional treatment aims, we implicitly acknowledge (at least in the given moment) that these aims are not obtainable; that is, to pursue them is futile.

This logic will lead to a host of ethical issues which are more traditionally associated with the even more contentious topic of palliative care in severe and long-standing AN and physician-assisted dying (PAD) for these patients [3]. Such ethical criticisms may refer to examples that exist of recovery from AN, even with severe levels of disease and protracted duration of illness [38]. The notion that treatment is no longer effective for patients with severe and long-standing AN has been well disputed in a 2018 paper by Raykos et al. which identified that traditional evidence-based interventions can have comparable effectiveness in chronic as well as acute patients [39]. Similar findings emerged from Dalle Grave and colleagues in an earlier 2017 study [40]. These observations were replicated in the UK by Ibrahim et al. [41]. With this in mind, we need to be careful of the blurry line in harm reduction between alleviating suffering and inadvertently reinforcing the patients’ psychopathology.

Furthermore, patients and caregivers may respond negatively to the concept that their condition is “treatment refractory”, and Elwyn gives an excellent account of how this label can in some cases generate rather than alleviate suffering, with concomitant effects on engagement with treatment [32].

It is clear from a range of evidence that for many patients, recovery is a continuous process, which may take years. Constructing a binary narrative that confines patients to being either recovered or refractory (and therefore deemed futile) could prove detrimental to how we approach this severe and long-standing AN. Similarly, other misleading binaries, such as those between early intervention and long-standing illness, and clinicians and patients themselves, should be avoided.

### Is a rejection of harm reduction throwing the baby out with the bathwater?

The above critique on three domains of the ethics of harm reduction should not cause us to dismiss the utility of this approach in certain contexts. Rather than harm reduction being a focus in and of itself, it may have utility when considered as part of a broader treatment pathway for a non-consenting patient, which still leads ultimately towards optimal treatment goals as its end point. Whilst the question of specificity as to when and for whom harm reduction may be useful remains to be resolved, a recent piece from Russell’s group has been particularly insightful in considering harm reduction within a wider context [16].

As with many interventions—be they psychological, family based, or pharmacological -studying them in isolation can lead to falsely narrow narratives that detract from the reality that recovery from ED is multidisciplinary and often occurs in phases [16, 42]. As Russell points out, it may be best to consider harm reduction as promoting the initial phase of recovery, which can then open the door for further recovery and ultimately weight restoration. Indeed, an excellent qualitative piece in recovered patients speaks of a recovery “tipping point”, whereby patients could escape from a repeated cycle of recovery and relapse by finding a new intrinsic motivation through gradual change and acceptance [42]. Furthermore, the emphasis harm reduction efforts place on quality of life may provide patients with a taster of a life worth recovering for, and more trusting relationships with healthcare professionals with whom alliance will be an essential ingredient of change-focussed evidenced-based therapies [36].

To this end, we would be interested in seeing further research conceptualising harm reduction as part of a step-wise model of recovery, and suggestions for what this could look like that are co-produced with patients. However, our tenet is that risks of a harm reduction approach should always be communicated to the patient, and the potential benefits harnessed as part of an overarching goal of full weight restoration and recovery, not the endpoint of treatment. 

## Conclusions

It is thought provoking to consider that in the 10 years between the first 2012 systematic review and the 2023 Cochrane review, there has been little progress in better *defining*, let alone developing, specific treatments for severe and long-standing AN. The ongoing lack of focussed treatment options has led to understandable pessimism, elsewhere described as a “therapeutic stagnation” in the field [6]. Clinicians doing their best with the limited resources available to them can be forgiven for reaching for harm reduction as a partial solution, albeit one still lacking a robust evidence base and ethical framework which may require years of further research to establish.

Demoralising as this progress may seem, recent findings of a large-scale meta-analysis have provided grounds for cautious optimism, given that even with the status quo, recovery rates amongst patients with AN were found to improve over the longer term [6]. The trend that has been demonstrated of recovery occurring later in life should provide an important motivation for clinicians and patients alike to engage with existing recovery-oriented approaches, and should stimulate ongoing research to establish better, more nuanced understandings of the role of harm reduction within this.

Indeed, our understanding of AN as a whole may be shifting towards a metabo-psychiatric diagnosis, with a recent genetic analysis from Watson’s group identifying several loci important in glycaemic control and lipid metabolism as being strongly associated with AN [43]. This “paradigm shift” could break new ground on what predisposes certain individuals to developing severe and long-standing presentations of AN, inspiring much-needed novel treatment innovations [44].

Further research will be crucial. This may include conducting longitudinal cohort studies to compare quality of life, morbidity, and mortality between patients receiving harm reduction therapies and those undergoing recovery-focused treatments. Additionally, economic evaluations are needed to assess the cost-effectiveness of harm reduction versus traditional methods, considering both direct and indirect healthcare costs over time. A mixed-methods study should also explore the emotional and practical impacts on patients and their families, shedding light on the social and familial consequences of different interventions. Such research could enhance our understanding of ethical management practices for this patient group.

Irrespective of future directions, it is essential to re-emphasise the potential risks associated with viewing harm reduction as the ultimate goal of treatment in severe and long-standing AN. Services must be willing to examine their motivations for using a harm reduction as an approach, the range of problems they are trying to balance when designing care pathways for their patients, and the ethical implications of treatment options for patients and their carers. As we hope to have demonstrated in the authoring of this article, collaboration with patients and carers enhances our understanding, and is achievable. We must be curious and honest about whose best interests' clinical decisions are made in, and whether harm reduction is more about removing intrapersonal and interpersonal conflicts that can arise within treatment, rather than removing harm itself.

## Data Availability

Data sharing is not applicable to this article as no new data were created or analysed in this study.

## Abbreviations

ED: Eating disorders
AN: Anorexia Nervosa
SEED: Severe and enduring eating disorders
ICD-11: International classification of diseases 11th revision
DSM-5: Diagnostic and statisitical manual of mental disorders 5th edition
SE-AN: Severe and enduring Anorexia Nervosa
BMI: Body Mass Index
RCT: Randomised controlled trial
CBT-AN: Cognitive behavioural therapy for Anorexia Nervosa
CBT-E: Enhanced cognitive behavioural therapy
COPP: Community outreach partnership program
SSCM: Specialist supportive clinical management
CRT: Cognitive remediation therapy
PAD: Physician assisted dying
JD: James Downs
AA: Agnes Ayton
EB: Edwin Birch
